# A Water-Soluble Polysaccharide from *Lophatherum gracile* Brongn.: Structure Characterization and Antitumor Activity In Vivo

**DOI:** 10.3390/foods15081300

**Published:** 2026-04-09

**Authors:** Xiaojing Zhang, Huizhen Xing, Huiping Liu, Xiaowei Zhang

**Affiliations:** State Key Laboratory of Food Nutrition and Safety, Ministry of Education of China, College of Food Science and Engineering, Tianjin University of Science and Technology, 13th Road, Economic and Technical Development Zone, Tianjin 300457, China; 18822269225@163.com (X.Z.); xinghuizhen1998@163.com (H.X.); zhangxw@tust.edu.cn (X.Z.)

**Keywords:** *Lophatherum gracile* Brongn., polysaccharide, structure, antitumor activity

## Abstract

*Lophatherum* *gracile* Brongn. (*L. gracile*) has been utilized as a food or medicinal plant for a long time. A series of chemical and spectroscopic methods was used to characterize the extracted and purified *L. gracile* polysaccharide (LGP). Its in vivo antitumor activity in the H22 tumor-bearing mice model was studied. LGP has a molecular weight of 1.42 × 10^6^ Da and is mainly composed of arabinose (Ara), galactose (Gal), xylose (Xyl), and other monosaccharides. NMR spectra suggest that LGP may be composed of 1,3-β-Gal*p* and 1,3,6-β-Gal*p* main chains, and a side chain formed by a 1,5-α-Ara*f* short chain. The termini are composed of T-α-Ara*f*, while [→4) -α-Gal*p*A-(1→2)-α-Rha*p*-(1→] are attached to the backbone as short side chains, and the other monosaccharides are an arabinogalactan composed of the termini. SEM and AFM revealed that LGP presents a lamellar morphology with smooth surfaces and notable molecular aggregation. The Congo red assay, CD spectroscopy, and XRD collectively indicated the absence of a triple helix conformation and an overall amorphous structure in LGP. Compared with the model group, LGP treatment improved body responses, immune organs, and SOD and MDA levels. The tumor cell apoptosis rate in the high-dose LGP group was 50.0%. In the distribution of the tumor cell cycle, the proportions of the S phase were 29.1% and 41.1% in the low-dose LGP and high-dose LGP groups, respectively, compared with 12.2% in the model group. These results suggest that LGP exhibits preliminary antitumor activity, indicating its potential as a candidate for further cancer research.

## 1. Introduction

Hepatocellular carcinoma (HCC) is one of the world’s most common cancers and has a high mortality rate [1,2]. The current main treatment methods or hepatocellular carcinoma include interventional therapy, molecular targeted therapy, and adjuvant therapy [3]. However, these treatments have limited clinical benefits and are associated with serious side effects and high costs [4]. Therefore, new safe and effective drugs against tumors are urgently needed. Polysaccharides usually refer to macromolecular substances comprising 10 or more monosaccharides connected by glycosidic bonds that are widely found in plants, animals, and microorganisms [5,6]. Depending on their source, polysaccharides can be categorized as animal, plant, or fungal polysaccharides [7]. Many studies have shown that different plant polysaccharides possess low toxicity and high biological activity [8]. Among them, leaf polysaccharides have attracted considerable attention. Peng et al. prepared two polysaccharide fractions from Camellia fascicularis leaves, both of which showed antioxidant and antitumor activity in vitro [9]. Li et al. extracted a high molecular weight polysaccharide (MLP-2C) from mulberry leaves, which demonstrated cholesterol-lowering effects [10].

*Lophatherum gracile* Brongn. (*L. gracile*), a Poaceae family (“Danzhuye” in Chinese) is widely found in southern China [11]. According to the *Bencao Gangmu* (*Herbal Foundation Compendium* published in Nanjing (China) in 1596), *L. gracile* has a long history in traditional Chinese medicine, being used to treat fever, anti-inflammatory, and other related diseases [12]. Modern pharmacological studies have shown that *L. gracile* exhibits biological activities such as antitumor, antioxidation, antivirus, anti-inflammatory, and liver-protective effects [11,13,14]. *L. gracile* is often used to make functional foods, such as teas and wines. For example, Zhuyeqing liquor (a functional drink mixed with *L. gracile*) has been reported to enhance immune activity in immunosuppressed mice [15]. Lai et al. demonstrated that the ethanol extract of *L. gracile* exhibited anti-inflammatory effects through the inhibition of JNK activation and calcium mobilization [12]. He et al. reported that an ethanol extract of *L. gracile* may protect against CCl_4_-induced liver injury by augmenting antioxidant enzymes [11]. Kim et al. demonstrated that an ethanol extract of *L. gracile* could inhibit the metastatic and angiogenic abilities of highly malignant HT1080 cells and had a superior therapeutic effect on the growth of intraovary transplanted tumors and lung metastasis in mice [13]. *L. gracile* is rich in flavonoids and polysaccharides. Polysaccharides with strong antioxidant and antitumor effects have been increasingly valued by researchers [16,17]. A growing body of research has highlighted the significant potential of polysaccharides from natural products as both medical and functional foods. However, studies on the polysaccharides of *L. gracile* have hitherto focused primarily on extraction methods and in vitro antioxidant activity. Further investigation is required into their fine structure and antitumor activity.

The antitumor activity of natural polysaccharides usually exhibits systemic regulatory effects, and its main mechanism is often achieved by activating the host’s immune system (e.g., enhancing macrophage phagocytosis and promoting cytokine secretion). In vitro cell lines lack a complete immune microenvironment, hindering the simulation of this multidimensional drug’s action process. The H22 cell line is derived from a mouse primary liver cancer and shares certain biological similarities with HCC, including its growth characteristics, pathological manifestations, and responses to immunomodulators [18]. In the present study, the structural characteristics of *L. gracile* polysaccharides were preliminarily characterized, and their therapeutic potential for HCC was investigated using an in vivo H22 mouse model. These results provide preliminary insights into the structural features of plant-derived polysaccharides with antitumor activity, suggesting their potential as natural candidates for further exploration in HCC intervention.

## 2. Materials and Methods

### 2.1. Materials and Reagents

*L. gracile* was bought from a local pharmacy in Tianjin and produced in Zhejiang Province (China) and stored in the laboratory of the College of Food Science and Engineering, Tianjin University of Science and Technology. Full botanical plant names have been checked and confirmed at http://powo.science.kew.org (accessed on 2 April 2026). Sephadex G-200 was obtained from Yuanye Biotechnology Co., Ltd. (Shanghai, China). T-series dextran, monosaccharide specimens, and H&E staining kits were acquired from Solarbio Ltd. (Beijing, China). H22 hepatocellular carcinoma cells were obtained from Shanghai Institutes for Biological Sciences. The Annexin V-FITC/PI apoptosis assay kit and cell cycle assay kit were supplied by Beyotime Biotechnology Co., Ltd. (Shanghai, China). The SOD and MDA kits were provided by Jiancheng Biochemical Reagent Co., Ltd. (Nanjing, China).

### 2.2. Preparation of LGP

The dried *L. gracile* was crushed into powder, sifted through a 60 mesh sieve, and degreased by Soxhlet extraction. The treated powder was placed in a beaker, mixed with distilled water (*v*:*w* 1:30), and extracted at 100 °C for 3 h. Then, the supernatant was obtained by centrifugation (7104× *g*, 15 min). The supernatant was concentrated to 400 mL using a rotary evaporator, 1.5 times absolute ethanol was added, and the mixture was left at 4 °C for 15 h. The precipitation was retained by centrifugation (7104× *g*, 15 min), then redissolved with distilled water. The ethanol was removed by spin distillation, the protein was removed by the Sevag method [19], and then dialysis was performed in distilled water with a dialysis bag (M_W_ cut-off 10 KDa) for 3 days. The dried material was a crude polysaccharide, which was purified by passage through a Sephadex G-200 gel column (1.6 × 40 cm; 2 mL/tube, 6 min/tube); the tube with the highest peak on the elution curve was collected, recovered, and freeze-dried to obtain LGP. HPLC analysis was performed under the same detection conditions as in Section 2.3.2.

### 2.3. Structural Characterization of LGP

#### 2.3.1. Chemical Composition

Total sugar content in polysaccharides was determined by the phenol–sulfuric acid method [20]. The glyoxylate content of the polysaccharide was obtained via the m-hydroxybenzene method [21]. The Coomassie Brilliant Blue method was used to determine the protein content of the polysaccharides. The nucleic acid and protein content of LGP was determined using UV–Vis spectrophotometry (Spectrum 2102-UV; American Thermal Sciences Inc., Wixom, MI, USA) in the 190-400 nm range.

#### 2.3.2. Molecular Weight (M_W_)

The M_W_ of LGP was determined by HPLC (Agilent-1200, Santa Clara, CA, USA). For this, 20 μL of 1 mg/mL LGP was collected with the instrument and eluted with ultrapure water at 0.6 mL/min with the column temperature set to 30 °C and the refractive index detector temperature to 35 °C. The retention time of glucan standards was used to make the standard curve, and the M_W_ of LGP was calculated [22].

#### 2.3.3. FT-IR Analysis

FT-IR (Bruker VECTOR-22, Karlsruhe, Germany) was used to determine the major functional groups in LGP. LGP (1 mg) was mixed with potassium bromide (150 mg) and ground before being pressed into thin sheets. Thin sections were scanned at a range of 4000 to 400 cm^−1^ [23].

#### 2.3.4. Monosaccharide Composition

The composition of monosaccharides was detected by ion chromatography (Dionex ICS-2500, Thermo, Sunnyvale, CA, USA) and analyzed as described in previous studies [24]. For this, 3 mg of LGP was dissolved in 0.6 mL (0.2 mol/L) trifluoroacetic acid (TFA) in an oil bath tube and hydrolyzed in an oil bath at 110 °C for 3 h, followed by blowing out the TFA with N_2_. The hydrolysate was dissolved in ultrapure water (1 mL). Monosaccharide standards were selected as references [25], including fucose (Fuc), rhamnose (Rha), arabinose (Ara), galactose (Gal), glucose (Glc), xylose (Xyl), mannose (Man), ribose (Rib), galacturonic acid (GalA), and glucuronic acid (GlcA). Monosaccharide analysis was conducted using a Dionex ICS2500 ion chromatography system (Thermo, USA) equipped with a pulsed amperometric detector. Chromatographic separation was achieved on a PA20 column (150 mm × 3 mm) maintained at 30 °C. The mobile phase, consisting of 10 mM NaOH and 200 mM sodium acetate, was delivered at 0.5 mL/min.

#### 2.3.5. NMR Analysis of LGP

For the NMR analysis, 60 mg of LGP was dissolved in 0.5 mL of deuterium oxide. The ^1^H, ^13^C, COSY, and HSQC spectra of LGP were recorded with a Bruker Avance III 400 MHz spectrometer (Bruker, Karlsruhe, Germany) [26].

#### 2.3.6. Observation of the Microstructure

The surface microstructure of LGP was detected by scanning electron microscopy (SEM) (SU1510; Hitachi, Japan) with magnifications of 150×, 300×, and 500×. Briefly, the morphology was examined after a small amount of the sample was uniformly distributed onto a 0.5 × 0.5 cm conductive adhesive and sputter-coated with a thin gold layer.

For atomic force microscopy (AFM) characterization, a 2–3 μL aliquot of the 5 μg/mL LGP solution was deposited onto freshly cleaved mica surfaces and subsequently lyophilized for 12 h. The surface topography was then imaged using a Bruker AFM system (Bruker, Santa Barbara, CA, USA) at 25 °C, with data acquired over a 10 × 10 μm^2^ scanning area.

#### 2.3.7. Triple Helix Structure

Congo red and circular dichroism (CD) experiments were used to analyze the system following the LGP reaction with different concentrations of Congo red and NaOH to determine the presence of a three-stranded helix structure [27].

After mixing LGP (0.5 mg/mL) with a Congo red solution (50 μmol/L), various volumes of a NaOH solution (1 mol/L) were added to create a NaOH concentration gradient ranging from 0 to 0.4 mol/L. After a 15 min reaction period, the maximum absorption wavelength was determined by scanning with an UV–visible spectrophotometer over 400–600 nm, with the Congo red–NaOH mixture at the corresponding concentration serving as the control.

LGP (0.5 mg/mL) was mixed with a Congo red solution (50 μmol/L) at the same volume. Then, the NaOH solution was added to achieve a final concentration of 0.5 mol/L. After 10 min, distilled water was used as a blank control, and the CD spectrometer scanned the wavelength range of 190–260 nm.

#### 2.3.8. X-Ray Diffraction (XRD)

The LGP was scanned and analyzed using an X-ray diffractometer [28]. The following scanning parameters were used: a scanning speed of 2°/min, and a 2θ range of 5–80° (Bruker D8, Bruker., Madison, WI, USA).

### 2.4. In Vivo Antitumor Activities of LGP

#### 2.4.1. Design of Mouse Models

As reported in previous studies [29,30], the mice were selected and purchased from SPF Biotechnology Co., Ltd. (Beijing, China, SCKX Beijing 2019-0010). All animal experiments were approved by the Ethics Committee of Tianjin University of Science and Technology (approval number: 2022025) and performed following the ARRIVE guidelines. During the experiment, the room’s humidity was maintained at 45–55% and the temperature at 22 ± 2 °C, and a 12 h circadian cycle was set. These conditions ensured that the mice ate and drank normally each day.

After one week of environmental adaptation, 10 mice were randomly selected as the blank group. The remaining 40 mice received a 0.2 mL injection of an H22 hepatocellular carcinoma cell suspension (1 × 10^6^ cells/mL) into the axilla of the left forelimb. These mice were then randomly assigned to the model, 5-Fu, low-dose (100 mg/kg), and high-dose (300 mg/kg) groups. No significant difference in initial tumor volume was observed among the groups. The LGP dose was based on pre-experiments and references to similar antitumor doses of plant-derived polysaccharides [17,31]. The blank and model groups received 0.2 mL of normal saline by gavage. The 5-Fu group received 5-fluorouracil (20 mg/kg) by intraperitoneal injection [17,32]. The low- and high-dose groups received LGP (0.2 mL, 100 mg/kg or 300 mg/kg) by gavage. The process lasted 15 days. Physiological parameters were continuously monitored. Tumor weighing, pathological section observation, and data analysis were performed by two independent, blinded researchers each to eliminate subjective bias.

#### 2.4.2. Tumor Weight and Tumor Inhibition Rate

After fasting for 12 h, the mice were sacrificed and dissected. The intact H22 solid tumors were excised, washed with sterile saline, and weighed. The tumor inhibition rate (TIR) was determined according to a previous study [31].

#### 2.4.3. The Immune Organ Index

During dissection, the thymus and spleen of the mice were retained and weighed accurately. The organ index was determined as the organ weight/body weight ratio in mice.

#### 2.4.4. Determination of MDA and SOD in Mouse Livers

The livers of the mice were homogenized in normal saline for 3 min (ratio:1 g/9 mL) and centrifuged (2000× *g*, 20 min), and the supernatant was collected. SOD and MDA ere determined using a commercial kit.

#### 2.4.5. H&E Staining

The liver tissue and solid tumor tissue grinding fluid were stained with an H&E staining kit and their morphological characteristics were observed with a microscope [33].

#### 2.4.6. Annexin V-FITC/PI Staining for Apoptosis Analysis

The tumor tissue was ground and washed through a cell sieve with PBS to obtain a tumor cell suspension, which was then centrifuged (2000× *g*, 5 min) at 4 °C. The solid tumor cell precipitate was resuspended with a binding buffer, and the staining solution was added according to the kit’s operation instructions for the reaction. Finally, the stained samples were examined using flow cytometry after passing through a 300 mesh cell screen.

#### 2.4.7. Cell Cycle Assay

According to the instructions of the kit, 1 mL of the cell suspension was fixed, and the staining solution was added for incubation. The cell cycle was measured by flow cytometry [16].

### 2.5. Statistical Analysis

SPSS 21.0 software was used to analyze the data, and the experimental data were expressed as the mean ± SD. One-way ANOVA was used for group comparisons, and Duncan’s multiple range test was used for multiple comparisons; *p* < 0.05 was considered to be statistically significant.

## 3. Results and Discussion

### 3.1. Chemical Composition of LGP

**Figure 1:**
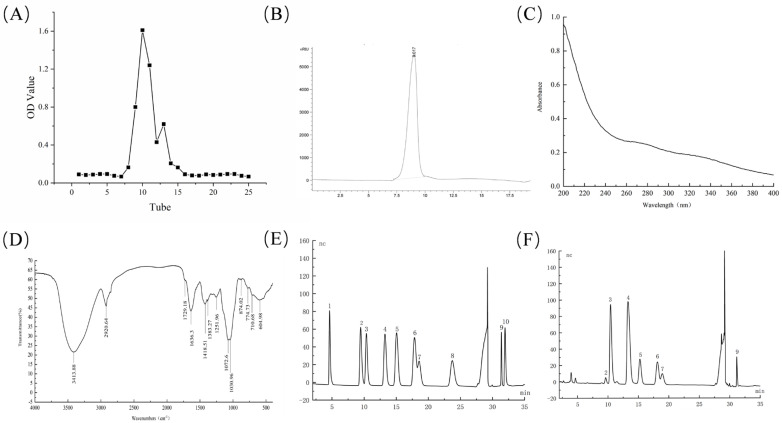
Elution profile (**A**), HPGPC chromatogram of LGP (**B**), UV spectrum of LGP (**C**), FT−IR (**D**), monosaccharides IC analysis of standards (**E**), and LGP (**F**). (1) Fucose; (2) rhamnose; (3) arabinose; (4) galactose; (5) glucose; (6) xylose; (7) mannose; (8) ribose; (9) galacturonic acid; (10) glucuronic acid.

The yield of the crude polysaccharide was 4.54 ± 0.26%. The elution curve of the Sephadex G-200 is shown in Figure 1A, and the major components were collected for detection. On the HPLC spectrum of LGP (Figure 1B), only a narrow symmetric peak appears, indicating a single and uniform distribution, which was freeze-dried and named LGP, with the yield being 1.02 ± 0.14%. The regression equation of the M_W_ was y = −0.3216x + 8.7973 (R^2^ = 0.9989). Based on the retention time (9.017 min), the M_W_ of LGP was 1.42 × 10^6^ Da.

The total sugar, protein, and uronic acid contents of LGP were 93.40%, 1.71%, and 5.50%, respectively. There were no signals at 260 nm and 280 nm (Figure 1C), indicating that nucleic acids and proteins were absent from LGP.

### 3.2. FT−IR Analysis

The FT-IR spectra of LGP are shown in Figure 1D. A broad absorption peak appeared at 3413.88 cm^−1^, which was O-H stretching [34]. The characteristic peak at 2920.64 cm^−1^ was caused by the C−H tensile band [35]. The 1729.18 cm^−1^ was related to the presence of uronic acid but was relatively weak, consistent with the detection result of uronic acid in Section 3.1. The asymmetric stretching of C=O can explain the peak at 1636.30 cm^−1^ [36]. The bending of C-H bonds at 1418.51 cm^−1^, 1338.27 cm^−1^, and 1251.96 cm^−1^ can be explained by the absorption peaks at these values [37], and the peaks at 1072.60 cm^−1^ and 1030.96 cm^−1^ suggested the presence of pyranoside [38].

### 3.3. Monosaccharide Composition

The composition of LGP was analyzed by ion chromatography and then compared with standard products. The retention time of standard products (E) and fully hydrolyzed products of LGP (F) is shown in Figure 1. LGP was composed of Rha, Ara, Gal, Glc, Xyl, Man, GalA, and GlcA, and the mole ratio was 0.12: 2.45: 3.18: 0.78: 1.00: 0.43: 0.24: 0.02. The Gal:Ara ratio of about 1.3:1 is characteristic of arabinogalactan. The ratio of GalA:Rha was approximately 2:1. This composition suggests that LGP may be a complex molecule, with arabinogalactan forming the main backbone to which other neutral sugar side chains are coupled, along with a small number of acidic domains.

### 3.4. NMR Results of LGP

**Figure 2:**
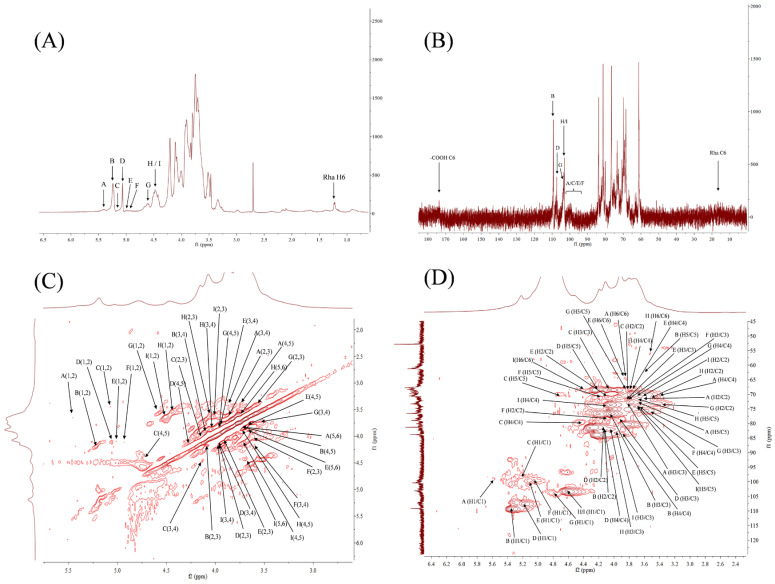
^1^H (**A**), ^13^C (**B**), ^1^H-^1^H COSY (**C**) and ^1^H-^13^C HSQC (**D**) NMR spectra of LGP.

The 1D (^1^H, ^13^C) and 2D (^1^H-^1^H COSY, ^1^H-^13^C HSQC) NMR spectra were used to analyze the fine structure of LGP comprehensively. Generally, the proton signal of the α-glycoside residues appears in the ^1^H NMR spectrum between 5.0 and 5.9 ppm, whereas the proton signal of the β-glycoside residues appears between 4.3 and 5.0 ppm [39]. The chemical shift of D_2_O was 4.79 ppm. The ^1^H NMR spectrum of LGP (Figure 2A) shows eight signal peaks at 5.35, 5.19, 5.06, 5.01, 4.94, 4.88, 4.56, and 4.43 ppm. In the ^13^C NMR spectrum, the signal from 98 to 103 ppm usually indicates an α-sugar residue, while a signal from 103 to 106 ppm indicates a β-sugar residue [40]. The ^13^C NMR spectrum of LGP (Figure 2B) showed prominent signal peaks at 109.37, 107.40, 104.06, and 103.39 ppm and weak peaks at 98–101 ppm. Additionally, the peak at 171.39 ppm indicates a small amount of uronic acid in LGP [41], consistent with previous analyses.

The ^1^H-^13^C HSQC spectrum (Figure 2D) and the ^1^H-^1^H COSY spectrum (Figure 2C) were used to assign the heterohead atom pair (H-1/C-1) exactly. The results are summarized in Table 1. Here, 5.35/98.97 ppm corresponds to T-α-Glc*p* (Residue A), 5.19/109.37 ppm corresponds to T-α-Ara*f* (Residue B), 5.06/98.57 ppm corresponds to 1,4-α-Gal*p*A (Residue C), 5.01/107.40 ppm corresponds to 1,5-α-Ara*f* (Residue D), 4.94/100.65 ppm corresponds to T-α-Man*p* (Residue E), 4.88/99.50 ppm corresponds to 1,2-α-Rha*p* (Residue F), 4.56/104.06 ppm corresponds to T-β-Xyl*p* (Residue G), and 103.38 ppm corresponds to 1,3-β-Gal*p* and 1,3,6-β-Gal*p* (residue H/I). However, 1.21/17.50 ppm was attributed to the H6/C6 of Residue F.

The combined monosaccharide composition and NMR spectra suggest that the LGP may be linked linearly via 1,3-β-D-Gal*p*, forming a rigid backbone with a structure similar to that of Type I arabinogalactan (AG-I). Some β-Gal*p* residues on the backbone were substituted at Position O-6 to form a 1,3,6-β-D-Gal*p* branch point. Short chains of 1,5-α-L-Ara*f* (residue D) are attached at Position C-6 of the branch point and possibly at position C-6 of the backbone portion, with T-α-Ara*f* (Residue B). This may be the main reason for the complexity and water solubility of LGP. Various terminal sugar residues, such as β-D-Xyl*p* (Residue G), α-D-Glc*p* (Residue A), and α-D-Man*p* (Residue E), can act as small side chains or caps that are directly attached to the free hydroxyl group of the main Gal*p* chain or to the end of the Ara*f* chain. This increases the microscopic heterogeneity of the structure. A small number of 1,4-α-Gal*p*A (C) and 1,2-α-Rha*p* (F) residues may exist as disaccharide units or as independent short chains attached to the main structure.

### 3.5. Observation of the Microstructure

**Figure 3:**
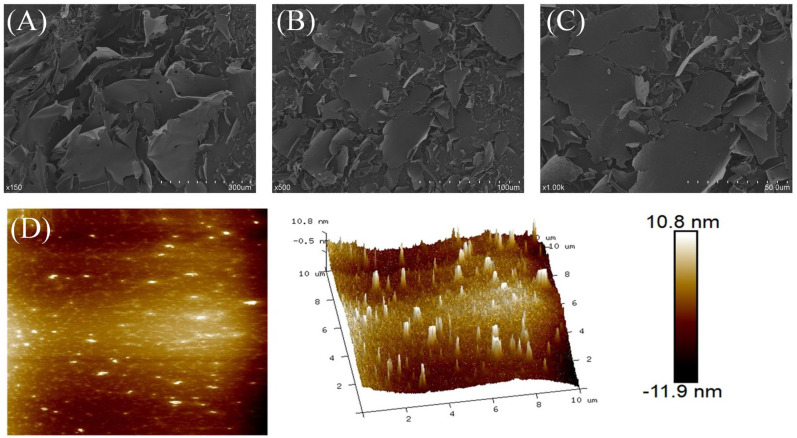
Morphology of LGP. SEM images (150× (**A**), 500× (**B**), 1000× (**C**) and AFM images (**D**).

The surface morphology of LGP under magnifications of 150×, 300× and 500× was observed by SEM. Figure 3A–C shows that the LGP has a lamelliform structure with a relatively smooth surface and less debris, and the lamella are closely connected and superimposed on each other.

The height of a single polysaccharide chain in AFM is generally between 0.1 and 1 nm. In contrast, the height of an LGP is between −11.9 and 10.8 nm (Figure 3D). This may be due to intermolecular interactions causing the polysaccharide molecules to aggregate and form polymers.

### 3.6. Triple Helix Structure

**Figure 4:**
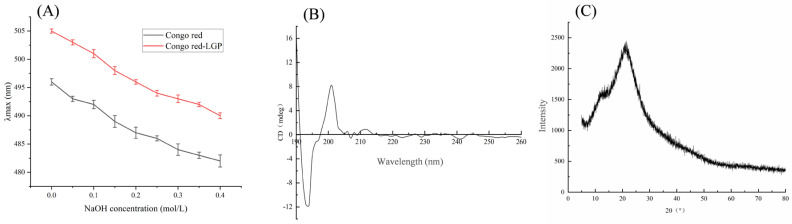
Congo red (**A**), CD analysis (**B**), and XRD (**C**).

Figure 4A shows that the maximum absorption wavelength of the LGP–Congo red solution mixture did not red shift and exhibited a downward trend as the NaOH concentration increased. This was consistent with the trend observed in the Congo red solution alone, indicating that the two compounds did not form a characteristic complex. This suggests that LGP does not have a triple helix structure.

CD analysis further revealed a negative Cotton effect at 190–200 nm and a positive Cotton effect at 201–205 nm, as shown in Figure 4B, inconsistent with the expected signal of a triple helix structure. These results were consistent with those of the Congo red experiment and confirmed that there was no triple helix conformation of LGP.

### 3.7. X-Ray Diffraction (XRD)

Figure 4C shows that the LGP exhibits a broad diffraction peak near 20°, with the remaining diffraction signals being weak. This diffraction feature indicates that the LGP has an overall amorphous structure.

### 3.8. In Vivo Antitumor Activities of LGP

#### 3.8.1. Effect of LGP on Body Weight and Solid Tumors in Mice

Table 2 shows the body weights of the mice throughout the experimental period. Initially, the five groups of mice had a consistent body weight of approximately 20 g. The loss of appetite and bradykinesia in the model group suggested that solid tumor growth had a negative effect on body weight and mental state. The weight of mice in the LGP group was significantly increased (*p* < 0.05) over that in the model group, and the mice survived well without death [42].

**Figure 5:**
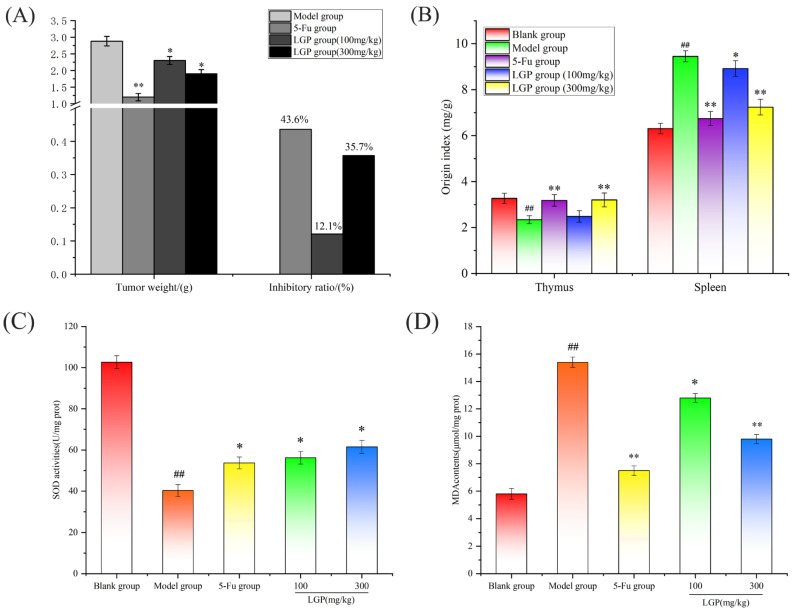
Tumor weights and inhibitory ratios of H22 tumor-bearing mice (**A**). Thymus and spleen organ indices (**B**). Determination of the levels of SOD (**C**) and MDA (**D**) in mouse livers.   ^##^ *p* < 0.01 for the model group vs. blank group; * *p* < 0.05, ** *p* < 0.01 for other groups vs. the model group.

The weight of the solid tumors in mice is shown in Figure 5A. It was calculated that the tumor inhibition rates were 12.1% and 35.7% for the 100 mg/kg LGP group and the 300 mg/kg LGP group, respectively, indicating that LGP can inhibit the growth of H22 tumor cells. In contrast, the 5-Fu group had the lowest tumor weight, with a tumor inhibition rate of 43.6%, showing that it could effectively inhibit tumor growth, but combined with the body weight and general behavioral characteristics of the mice, it was known that 5-Fu had serious side effects.

#### 3.8.2. Effects of LGP on Immune Organs

The thymus and liver are essential for immunity [43,44,45]. The organ indices of the thymus and spleen in mice are shown in Figure 5B. A significant reduction in the thymus index (*p* < 0.05) and a significant elevation in the spleen index (*p* < 0.05) were observed in the model group as opposed to the blank group. The mice in the model group exhibited thymus atrophy and spleen enlargement, showing that the tumor would damage the immune organs. The organ indices of the LGP group were improved, showing that LGP could protect the immune organs of mice.

#### 3.8.3. Determination of the Levels of SOD and MDA in the Liver of Mice

SOD is an antioxidant enzyme that scavenges superoxide radicals and is crucial in preventing oxidative damage [46]. MDA is a lipid peroxide produced in the process of homeostasis, which can reflect the degree of damage caused by lipid peroxides in the body [47,48]. According to Figure 5C,D, the liver SOD activity in the LGP treatment group was significantly higher (*p* < 0.05) than that in the model group, and the MDA content was significantly lower (*p* < 0.05), indicating that LGP can improve the overall antioxidant defense ability of mice and protect liver tissue from oxidative stress, thus fighting tumors.

#### 3.8.4. H&E Staining

**Figure 6:**
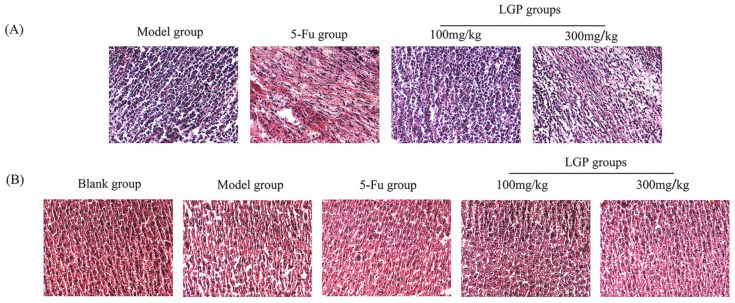
H&E staining of murine solid H22 tumors (**A**) and liver tissues (**B**).

Figure 6A shows that the tumor cells in the model group mice were numerous, morphologically complete, and closely arranged, and their nuclei were uniformly stained blue. The tumor cells in the 5-Fu and LGP treatment groups became loosely arranged and showed varying degrees of necrosis. There were more apoptotic tumor cells in the 5-Fu and high-dose groups. With increasing LGP content, the degree of solid tumor cell damage was more severe, suggesting that LGP-induced damage to solid tumor cells may be dose-dependent.

According to the section of liver tissues (Figure 6B), the hepatocytes were tightly arranged, and the nuclear structure was clear. After inoculation with H22 hepatocellular carcinoma cells, the morphology of hepatocytes in the model group was damaged, with the cytoplasmic space enlarged. The degree of hepatocyte necrosis in the LGP group was significantly lower than that in the model group, and the difference was more obvious in the high-dose group. The liver tissue cells were damaged in the 5-Fu group, indicating that 5-Fu had certain side effects.

#### 3.8.5. Annexin V-FITC/PI Staining for Detection of Apoptosis

**Figure 7:**
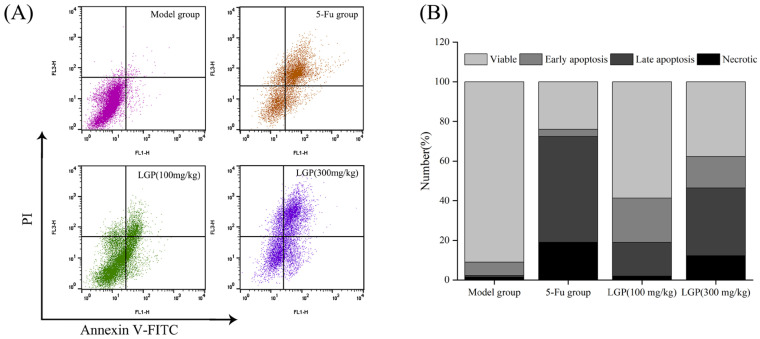
Apoptosis analysis of H22 solid tumor cells by Annexin V-FITC/PI staining. Histograms of the proportion of apoptotic cells by treatment group (**A**), and apoptotic proportions in solid tumors (**B**).

During early apoptosis, phosphatidylserine from the cell membrane is transferred to the membrane surface and binds specifically to the Ca^2+^-dependent phospholipid binding protein Annexin V [49]. In late apoptosis, the nucleus was stained red by pyridine iodide [50]. It can be seen from Figure 7 that 90.97% of the normal tumor cells in the model group were in good tumor condition. After LGP treatment, the apoptosis rate of the 100 mg/kg LGP group and the 300 mg/kg LGP group increased from 7.64% (model group) to 39.3% and 50% (*p* < 0.05), respectively, and the degree of tumor cell necrosis increased. The apoptosis rate in the 5-Fu group was 57%, and late apoptotic cells outnumbered early apoptotic cells. These results indicated that LGP could induce apoptosis of solid tumors of H22 mouse cells and inhibit proliferation in vivo.

#### 3.8.6. Effect of LGP on the Distribution of Solid Tumor Cells

**Figure 8:**
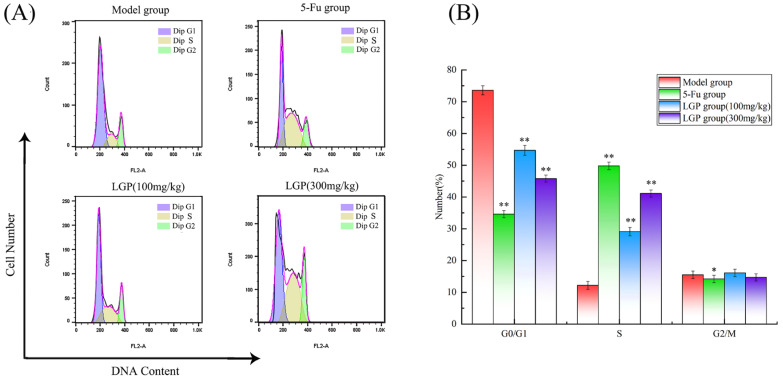
Cell cycle distribution of H22 solid tumor cells. Histogram of the DNA content of the treatment groups (**A**) and bar chart of the proportion of cell cycle phases G0/G1, S, and G2/M (**B**). * *p* < 0.05, ** *p* < 0.01 for other groups vs. model group.

Antineoplastic drugs are known to block the cell cycle at specific stages [51]. The tumor cell cycle distribution of H22 mice is shown in Figure 8. In the model group, the proportions of the G0/G1, S and G2/M phases were 73.6%, 12.2%, and 15.5%, respectively. The G0/G1 and G2/M phases in the LGP group were significantly less than those in the model group (*p* < 0.05), and the S phase was significantly increased, reaching 29.1% (100 mg/kg) and 41.1% (300 mg/kg), respectively. This result suggested that LGP could induce apoptosis in H22 cells, which was accompanied by an accumulation of cells in the S phase.

## 4. Discussion

Natural products are increasingly becoming the focus of research on new anticancer drugs due to their abundant sources, low cytotoxicity, and good antitumor effects. Edible plant polysaccharides are one of the most effective natural products for cancer prevention and treatment. GFP-A, a polysaccharide extracted from *Grifola* frondosa by Zhang et al., inhibited the growth of S180 tumor cells by enhancing immune activity and promoting the secretion of the cytokines TNF-α, IL-2, and INF-γ, inducing immune responses, and blocking the cell cycle [52]. Tao et al. extracted a polysaccharide from ginseng, which can significantly increase the phagocytosis function of macrophages [53].

The structural characterization of LGP provides a molecular basis for its remarkable antitumor activity in H22 tumor-bearing mice. The monosaccharide composition of LGP was mainly galactose (3.18), arabinose (2.45), and xylose (1.00), and the Gal/Ara ratio was about 1.3:1. Given the presence of Rha and GalA, it is strongly suggested that LGP may be an RG-I pectin structure decorated with arabinoglycoside side chains. Polysaccharides rich in galactosyl residues often display natural hepatotropic properties, and their exposed galactosyl domains enable ASGPR to bind to hepatocytes, thereby providing a targeted delivery mechanism for liver tumors [54,55]. Additionally, the high arabinose content suggests a dense, branched structure. As demonstrated by Jia et al. (2023) in the ginseng RG-I fraction, increased arabinose fractions enhanced anti-HCC potency, corroborating that side chain density determines receptor aggregation and cell surface affinity [56]. The presence of xylose and uronic acid indicates a complex heteropolysaccharide nature, which may further enhance sugar–protein interactions or disrupt lectin-mediated tumor adhesion, similar to the mechanism reported for *Lonicerae japonicae* [57]. Taken together, these structural features, including the degree of branching, the content of uronic acid, and the specific Gal/Ara ratio, suggest that LGP has a favorable antitumor effect. The linkage mode of LGP was initially inferred by chemical shift attribution of NMR and combined with the known studies of polysaccharides from the same genus. In future, the fine structure of LGP can be further verified by GC-MS.

The antitumor effect of polysaccharides is realized through inducing immune mechanism, inducing tumor cell apoptosis, and so on. Apoptosis is a programmed means of cell death activated by endogenous or exogenous signaling pathways. Research on the pathway of cancer cell death can reveal the main mechanisms and pathways leading to its death and provide relevant targets for cancer treatment [58]. Antitumor drugs generally block the cell cycle in a specific stage, thus causing apoptosis of tumor cells, and the main stage of DNA replication is the S phase [59]. The cell cycle analysis showed an increase in the proportion of cells in the S phase following LGP treatment, suggesting that the inhibitory effect of LGP on cell proliferation is associated with alterations in the cell cycle distribution. In this study, the body weight of the mice remained stable, and no obvious abnormal behavior was observed during the administration, which initially implied that LGP was well-tolerated at this dose. However, the strong effects on liver and kidney function still need to be further evaluated by serum biochemistry and associated histopathology. Further studies are needed to fully elucidate the fine structure and underlying molecular mechanisms.

## 5. Conclusions

This study investigated the structural characteristics of a novel high-molecular-weight polysaccharide (LGP) and evaluated its potential antitumor activity. Analysis of its structural features showed that the average molecular weight of LGP was 1.42 × 10^6^ Da and that it was mainly composed of Rha, Ara, Gal, Glc, Xyl, Man, and GalA, with molar ratios of 0.12:2.45:3.18:0.78:1.00:0.43:0.24. LGP may be a complex arabinogalactan consisting of a 1,3-β-D-Gal*p* main chain and a 1,5-α-L-Ara*f* side chain. Other neutral sugars are linked at the C-6 position, and the structure may contain traces of the characteristic pectin structure. The microstructure of LGP was examined using SEM and AFM. The results showed that LGP possesses a lamellar morphology with a smooth surface, and its molecular chains exhibit aggregation due to intermolecular interactions. Furthermore, analyses including a Congo red assay, CD spectroscopy, and XRD consistently indicated that LGP lacks a triple helix conformation and exhibits an amorphous structural nature. Furthermore, LGP was found to inhibit the growth of H22 hepatocellular carcinoma cells and exhibit a protective effect on the immune organs. Annexin V-FITC/PI double staining showed that LGP treatment increased the apoptotic rate of H22 cells. Additionally, PI staining indicated an accumulation of cells in the S phase, suggesting that the inhibitory effect of LGP is associated with alterations in the cell cycle distribution. LGP demonstrated a moderate inhibitory effect on tumor growth, which may provide a preliminary basis for its exploration as a natural functional ingredient in cancer intervention.

## Figures and Tables

**Table 1:** ^1^H and ^13^C NMR chemical shift assignment of sugar residues.

Glycosidic Bond		H-1/C-1	H-2/C-2	H-3/C-3	H-4/C-4	H-5/C-5	H-6/C-6
A	T-α-Glc*p*	5.35	3.64	3.85	3.41	3.75	3.86
		98.97	71.78	72.82	70.27	72.58	64.39
B	T-α-Ara*f*	5.19	4.15	4.07	3.90	3.70	
		109.37	81.67	76.61	84.11	63.31	
C	1,4-α-Gal*p*A	5.06	3.85	4.12	4.48	4.75	-
		98.57	67.57	68.55	79.84	70.72	171.39
D	1,5-α-Ara*f*	5.01	4.12	3.95	4.08	4.24	
		107.40	81.67	78.74	82.00	68.39	
E	T-α-Man*p*	4.94	4.10	3.90	3.77	3.66	3.90
		100.65	71.28	71.84	67.94	74.37	64.18
F	1,2-α-Rha*p*	4.90	4.10	3.90	3.60	4.20	1.21
		99.50	78.38	71.84	74.00	71.20	17.50
G	T-β-Xyl*p*	4.56	3.41	3.56	3.75	3.92	
		104.06	73.53	76.40	70.17	67.74	
H	1,3-β-Gal*p*	4.43	3.54	4.04	3.85	3.71	3.60
		103.38	71.39	82.00	68.58	72.12	59.98
I	1,3,6-β-Gal*p*	4.43	3.65	3.96	4.15	3.70	4.43
		103.38	70.72	82.84	76.79	74.37	68.66

**Table 2:** Effects of LGP on mouse body weight.

Group	Dosage (mg/kg/Day)	Body Weight (g)		Average Weight Increment (g)	Number of Mice (Start/End)
		Initial Weight	Final Weight		
Blank group	-	21.3 ± 0.50	31.84 ± 1.05	10.54 ± 0.78	10/10
Model group	-	20.55 ± 1.04	25.55 ± 0.81 ^#^	5.0 ± 0.52 ^#^	10/8
5-Fu group	20	20.5 ± 1.01	29.4 ± 0.99 *	8.9 ± 1.26 *	10/9
LGP group	100	21.25 ± 0.87	30.88 ± 0.99 *	9.63 ± 0.77 *	10/10
	300	21.57 ± 0.65	31.24 ± 1.05 *	9.67 ± 0.88 *	10/10

Note: ^#^ *p* < 0.05 model group vs. blank group; * *p* < 0.05 other groups vs. model group.

## Data Availability

The original contributions presented in this study are included in the article. Further inquiries can be directed to the corresponding author.

## Abbreviations

*L. gracile*	*Lophatherum gracile* Brongn.
LGP	*L. gracile* polysaccharides
HPLC	High-performance liquid chromatography
FT-IR	Infrared spectroscopy
NMR	Nuclear magnetic resonance
SEM	Scanning electron microscopy
AFM	Atomic force microscopy
CD	Circular dichroism
XRD	X-ray diffraction
Rha	Rhamnose
Ara	Arabinose
Gal	Galactose
Glc	Glucose
Xyl	Xylose
Man	Mannose
GalA	Galacturonic acid
GlcA	Glucuronic acid
H&E	Hematoxylin and eosin
SOD	Superoxide dismutase
MDA	Malondialdehyde
TFA	Trifluoroacetic acid
TIR	Tumor inhibition rate
